# Correlations between Dental Implant Infectious Pathologies and Maxillary Sinusitis: A Review Article

**DOI:** 10.3390/jcm12155059

**Published:** 2023-08-01

**Authors:** Simina Angela Lăcrimioara Iușan, Carmen Costache, Ondine Patricia Lucaciu, Bianca-Nausica Petrescu, Ioana Codruța Mirică, Dan-Alexandru Toc, Silviu Albu

**Affiliations:** 1 Department of Oral Health, Iuliu Hatieganu University of Medicine and Pharmacy, 400347 Cluj-Napoca, Romania; 2 Department of Microbiology, Iuliu Hatieganu University of Medicine and Pharmacy, 400347 Cluj-Napoca, Romania; 3 II-nd Department of Otolaryngology, Iuliu Hatieganu University of Medicine and Pharmacy, 400347 Cluj-Napoca, Romania

**Keywords:** dental implants, peri-implantitis, maxillary sinusitis, bone graft, sinus lift, oro-antral communication

## Abstract

(1) Background: The demands of patients for aesthetic and functional rehabilitation of edentulous areas led to the use of dental implants as therapeutic means on an increasingly large scale. This aspect determined the appearance of some infectious pathologies with a peri-implant starting point that can be complicated by various sinus diseases. The purpose of this review article is to synthesize the existing information in the specialized literature regarding the existing correlations between peri-implant and maxillary sinusitis. (2) Methods: The articles published in five databases were researched using different combinations of search terms. We selected 12 articles from the 250 found, by applying the inclusion and exclusion criteria and removing duplicates. (3) Results: We analyzed the included studies and we found that all of them reported a positive correlation between maxillary sinusitis and peri-implant infectious diseases. There are also reported other pathologies with a peri-implant infectious disease as a starting point such as abscesses, oro-antral communications, or foreign body reactions due to implant or bone graft materials migration. (4) Conclusions: This scoping review highlighted the existence of correlations between peri-implant and sinus pathology and the importance of preventing peri-implant diseases of an infectious nature to avoid the occurrence of these complications.

## 1. Introduction

Dental implants are increasingly used for oral rehabilitation of partially and fully edentulous areas [1]. This method is preferred by many physicians over conventional fixed or removable partial dentures because functionality and aesthetics are similar to those of natural teeth [2,3,4].

### 1.1. Dental Implants and Bone Evaluation

The technological evolution in dentistry and the use of 3D Cone Beam Computed Tomography (CBCT) helps dental surgeons to be more predictable. They can evaluate the bone quality and quantity before the surgery and properly plan the surgical treatment [4].

### 1.2. Bone Augmentation Techniques

There are many situations in which residual alveolar bone is deficient, especially in maxillary areas. Post-extraction continuous alveolar ridge resorption, pneumatization of the maxillary sinus after teeth loss, traumatisms, bone infections, and neoplasms reduce the bone availability for implant placement [2,5,6,7]. In addition, the lateral maxillary alveolar ridge is known as low-density medullar bone, and this is translated into the low qualitative and quantitative bone area [2]. To expand the indications of dental implants and to have good quality and quantity of the alveolar bone ridge in these areas for implant placement, sinus augmentation procedures are used to enhance bone height [2,6,7,8]. Sinus augmentation techniques use different materials such as autografts, allografts, and xenografts placed into the sinus cavity [7].

### 1.3. Sinus Complications of Surgical Intervention for Bone Augmentation and Implant Placement

Despite well-established surgical treatment protocols and analyses of the cases, there are many complications that occur after sinus elevation interventions and placement of implants in these areas: membrane perforation and the formation of an oro-antral fistula, acute and chronic rhinosinusitis, facial congestion, nasal obstruction, implant displacement into the maxillary sinus and sinus graft failure [2,6,7]. Maxillary sinusitis, or rhinosinusitis when associated with rhinitis, is an inflammation of the maxillary sinus mucosa and is an acute condition when the symptomatology lasts less than 12 weeks and chronic when it exceeds this period [9]. Rhinosinusitis can be classified as acute, sub-acute, recurrent, and chronic according to the signs, symptoms, and course of the disease [10]. Chronic rhinosinusitis is associated with nasal airway obstruction, mucus secretions, headache, facial pain, and anosmia [11]. Nasal obstruction associated with sinusitis impacts respiratory function and affects the patient’s well-being, representing a debilitating condition that requires treatment to restore airway patency [12]. In addition, there is another complication that occurred after the sinus lift augmentation intervention and implant placement: the evolution of the infection from an implant affected by peri-implantitis, to the grafted area into the maxillary sinus [6]. Mucositis and peri-implantitis are infectious diseases of the surrounding tissue of a functional dental implant. Both diseases are caused by bacterial biofilm formed around the intraoral components of dental implants [13,14]. The most common bacteria that cause peri-implant tissue inflammation are gram-negative bacteria such as *Porphyromonas gingivalis*, *Prevotella intermedia*, *Treponema denticola*, *Prevotella nigrescens*, *Fusobacterium nucleatum*, and *Peptostreptococcus* spp. [14]. While mucositis is defined as an inflammation of the peri-implant mucosa without bone loss, peri-implantitis is characterized by inflammatory signs of peri-implant tissues and bone loss after functional marginal bone remodeling [13]. The prevalence of these diseases are quite high, with a mean for peri-implantitis of 21.7% [13], so the correlation between peri-implantitis and sinusitis, and the modality of developing sinusitis with a peri-implantitis starting point is an important issue to be investigated by practicians and researchers. This review article aimed to identify the correlation between peri-implantitis and sinusitis in order to raise awareness about the importance of the protocol of surgical procedures.

## 2. Materials and Methods

### 2.1. Research Strategy

The methodological design of this study is in accordance with PRISMA criteria and guidelines [15]. The article is not listed in any systematic review registries. In this review article, we addressed the following question: What are the correlations between peri-implantar infectious diseases and maxillary sinusitis? To find out the answer for this question, a systematic review of the literature was performed by two independent co-authors, in five databases: PubMed [16], Embase [17], Web of Science [18], Scopus [19], and SpringerLink [20]. The search strategy involved different combinations of MeSH keywords: “peri-implantitis”, “peri-mucositis”, “peri-implant mucositis”, “sinusitis”, “maxillary sinusitis”: (peri-implantitis) and (sinusitis), (peri-implantitis) and (maxillary sinusitis), (peri-mucositis) and (sinusitis), (peri-mucositis) and (maxillary sinusitis), (peri-implant mucositis) and (sinusitis), (peri-implant mucositis) and (maxillary sinusitis). The following filters have been applied: Article, Case reports, Classical Article, Clinical Study, Clinical Trial, Randomized Controlled Trial, English, French. The authors reproduced the same search strategy previously described for each of the 5 databases. 

### 2.2. Inclusion and Exclusion Criteria

The inclusion criteria were articles published in the five above-mentioned databases, between 1 January 2000 and 1 January 2023. Among the exclusion criteria were articles published in languages other than English or French and studies performed on other species than humans. Furthermore, articles not available in full-text format, systematic reviews, and meta-analyses were also excluded. 

### 2.3. Study Selection and Data Collection

The study selection and data collection were performed by two reviewers and if there were any doubts about the inclusion of the studies, a third one participated in the decision-making process. The titles and abstracts of the selected articles were analyzed in relation to the proposed research topic and, subsequently, the articles considered eligible were fully evaluated based on the inclusion and exclusion criteria. In the first instance, 250 articles were selected from the five mentioned databases in accordance with the search terms previously mentioned. After the initial screening of the selected articles, 163 duplicates and 9 articles without full-text availability were identified and excluded. We did comprehensive research of all databases, libraries, links, and sites through institutional access to find out the full-text availability of the nine excluded articles. Subsequently, 78 articles were analyzed in detail based on the inclusion and exclusion criteria and in relevance to the subject. Out of 78 articles, 12 reviews were identified and eliminated, and 54 articles were excluded because they were not in accordance with the proposed research. After detailed analyses, 12 articles that met the eligibility criteria were selected and included in this review article.

### 2.4. Risk of Bias Assessment (Methodological Quality)

Since 11 out of 12 articles included in this review are case reports and case series, we used a modified version of the Newcastle-Ottawa scale (NOS) [21] to assess the methodological quality of these articles, because there are no available tools to quantify the risk of bias for case reports and case series. We removed from the NOS scale the elements related to comparability and adjustment (because the studies included in this review were non-comparative). We selected the items that focused on selection, representativeness of cases, and ascertainment of outcome and exposure to assess the risk of bias. The resulting tool is formed of five criteria in the form of questions with a binary response (yes/no). These questions are listed in Table 1. We considered the high methodological quality of the study (low risk of bias) when all five criteria were achieved, moderate methodological quality (moderate risk of bias) when four criteria were achieved, and poor methodological quality (high risk of bias) when three or fewer were achieved. This tool for risk of bias assessment has been previously mentioned in other publications [22,23,24,25,26,27]. For the other article included in this review, which is a case-control study, we used the NOS scale to assess the risk of bias, and the results are shown in Table 2. The NOS scale quantifies three quality characteristics (selection, comparability, and outcome), divided into eight specific items. Each category on the scale is scored with a maximum of one point, except for comparability, which can be scored with two points. The maximum qualifier that can be obtained for each study is 9 [21]. Studies with NOS scores 0–3 are considered as low quality and high risk of bias, scores 4–6 as moderate quality and moderate risk of bias, and scores 7–9 are considered as high quality and low risk of bias [21] The quality assessment was carried out by two authors and no disagreements were found between them.

## 3. Results

### 3.1. Studies Characteristics

**Figure 1:**
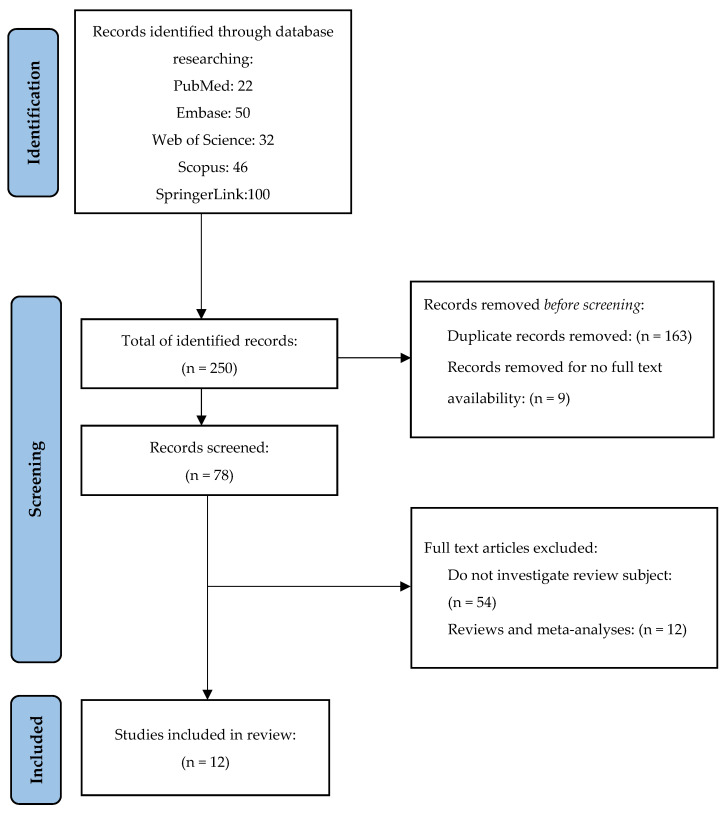
PRISMA flow diagram. Study selection for review [15].

The process of the literature search and study selection flow diagram are shown in Figure 1. We identified twelve publications between 2000 and 2023 that met the eligibility criteria and were included in this review. All of them are written in English and are available in full-text version. Regarding the quality of studies, five studies were appraised as high methodological quality and seven as moderate methodological quality as reported in Table 1 and Table 2. Concerning the type of studies included in this review, the publication date, and other information with reference to the patient data and the observed pathologies, are summarized in Table 3. Six articles out of twelve are case series, five articles are case reports, and one article is a case-control study.

### 3.2. Correlation among Pathologies

#### 3.2.1. Peri-Implantitis, Sinus Elevation with Bone Augmentation, and Chronic or Acute Sinusitis

In the studies included in this review, the most common association was between sinus elevation, peri-implantitis, and maxillary sinusitis. Seven studies [4,6,7,10,31,32,34] reported sinus complications after sinus graft techniques and implant insertion associated with peri-implantitis. 

Hainăroșie et al. [4] reported peri-implantitis and maxillary sinusitis as a complication in patients who received a sinus lift procedure concomitant with implant placement. Out of 21 patients, four developed peri-implantitis, and eleven developed acute maxillary sinusitis.

Won-Bae Park et al. [6] presented a case of a patient who underwent sinus augmentation with implant placement in his left posterior superior maxilla 15 years ago. Due to untreated peri-implantitis, an infection spread into the bone augmentation area and maxillary sinusitis occurred. 

Antonio Scarano et al. [7] presented a case series of 5 patients who received sub-antral augmentation and implant insertion after a healing period of 6 months. At 3 to 5 years post-loading, patients lost their implants due to peri-implantitis. All five patients were diagnosed with maxillary sinusitis after the removal of dental implants. The infection spread into the bone graft and has induced sinus graft infection. The bacteria were present inside the bone grafts, and the augmented area, newly formed bone was surrounded by necrotic tissues, inflammatory cells, and bacteria. 

Sung ok Hong et al. [10] reported a case of a 47-year-old man who developed acute maxillary sinusitis ten days after crestal socket osteotomy, bone graft, and implant insertion provoked by graft materials dissemination into the maxillary sinus. 

Won-Bae Park et al. [31] reported in a case series eight patients out of 124 who received implant placement with sinus floor augmentation and developed maxillary sinusitis associated with peri-implantitis.

Angel Emmanuel Rodriguez et al. [32] in a five-case series report the long-term risks and complications after sinus graft and implant insertion. The most frequent adverse effects presented in this case series were maxillary sinus and bone infections, displacement of the graft materials, oroantral communications, implant failure due to peri-implantitis, and soft tissue fenestrations. These complications occurred from 2 to 13 years after the initial treatment which was deemed successful.

Marn Joon Park et al. [34] in a case-control study compared implant-related complications between the case group of patients with implant-related odontogenic sinusitis (IR-ODS) and the control group of patients with dental implants and no clinical and radiographical signs of sinusitis. According to this study, peri-implantitis was significantly more frequent in IR-ODS sites compared to control sites. 

#### 3.2.2. Peri-Implantitis Sinusitis and Brain Abscess

Steiner C. et al. [28] reports a case of a 62-year-old man with a large brain abscess in the left frontal lobe, presenting severe headache and progressive aphasia. Sinusitis was present in the left maxillary and frontal sinuses. The authors identified as the source of infection untreated end-stage peri-implantitis on a dental implant in the left upper jaw in contact with the sinus floor. Despite the treatment applied patient died 10 months later. 

Hainăroșie et al. [4] in their study presents one case of orbital abscess and two cases of malar abscesses with the starting point of infection at an implant site.

#### 3.2.3. Peri-Implantitis and Oro-Antral Communications

Oro-antral communications are complications that can occur both in cases of dental implants and zygomatic implants.

Antonio D’Agostino et al. [5] reported two zygomatic implants with oro-antral communications out of 141 zygomatic implants analyzed in their study, and Mustafa Yalçın et al. [30] reported two cases of oro-antral communications in two patients out of the 42 who were included in their study.

Won-Bae Park et al. [6] in a case report presented a patient who developed sinusitis having as a starting point peri-implantitis around a dental implant in the premolar area. After removing the implant due to loss of osseointegration, an oro-antral communication was created and despite the closure of the communication, sinusitis persisted after implant removal and was solved with functional endoscopic sinus surgery in an otorhinolaryngology clinic.

Antonio Scarano et al. [7] reports also one case of oro-antral communication in an implant failure due to peri-implantitis. 

#### 3.2.4. Implants Migration/Bone Graft Extrusion into Maxillary Sinus and Sinusitis

Marn Joon Park et al. [34] in a case-control study reported that in cases of implant-related odontogenic sinusitis (IR-ODS) was observed a significantly higher frequency of bone graft extrusion into maxillary sinus compared to control sites, unaffected by sinusitis. Furthermore, implant exposure of more than 4 mm into the maxillary sinus was more prevalent in IR-ODS sites than in control sites. 

Hainăroșie et al. [4] reported 3 cases of patients with implant migration into the maxillary sinus, Van de Loo S et al. [29] presented one implant located under the left inferior nasal concha and Won-Bae Park et al. [31] reported one patient with an implant displaced into the maxillary sinus. 

#### 3.2.5. Zygomatic Implants and Sinusitis

There are many patients with upper maxilla extreme atrophy, trauma, cleft palate, or failed reconstruction treated with zygomatic implants. This type of implant needs an invasive surgical intervention which increases the risk of developing postoperative complications. In our review, we have included two articles [5,30] exposing the main complications related to zygomatic implant placement. 

Antonio D’Agostino et al. [5] in a retrospective evaluation with a five-year follow-up which included 42 patients with 116 zygomatic implants reported nine patients with sino-nasal complications, eight patients were confirmed with symptomatic sinusitis, two oro-antral communications and one patient with both, oro-antral communication, and sinusitis. 

Mustafa Yalçın et al. [30] in a retrospective analysis in which were included 45 patients with 141 zygomatic implants reported three patients who developed sinusitis. 

### 3.3. Extrapolation of the Result

Considering the high and moderate methodological quality assessment of all included studies in this review, the reports from eight different countries, the conclusions of this research could be applied in all cases of sinus pathology starting from the implant level.

## 4. Discussion

The world population is aging rapidly and according to the World Population Aging Report, the percentage of older people has been increasing considerably in recent years and continues to grow. In 2019, there were approximately 703 million people aged 65 years or above, and by 2050 the number of elderly people is expected to reach 1.5 billion [35]. The elderly population growing is accompanied by an increase in the number of patients suffering from dental diseases and indentations. Quality of life improvement in an aging society is an important issue and oral rehabilitation has a major contribution [35]. Dental implants are widely accepted as being the core of rehabilitation in edentulous patients [35,36] so their use in oral rehabilitation is increasing rapidly. The dental implants global market recorded 5% growth in 2020 and is expected to have an annual growth rate of 5.0% through 2026 [35].

Even though dental implants are widely applied, the lateral maxillary area can bring challenges regarding their insertion. The resorption of the maxillary bone ridge in the lateral area, caused by alveolar bone resorption and pneumatization of the maxillary sinuses, leads to the quantitative decrease of the maxillary bone available for the insertion of dental implants. In these situations, to facilitate implant insertion, a reconstructive phase with a bone augmentation sinus lift procedure is mandatory [37,38,39].

Although dental implants offer a predictable solution for missing tooth replacement, and their survival rates are high, failure could occur as a multifactorial problem [40]. Implant loss could appear during the osseointegration process (early implant loss) or at a time point after, when the achieved osseointegration is lost (late implant loss) [41]. Early failure is associated with various causes such as contamination during surgery, overheating, trauma, poor bone quantity and/or quality, incorrect immediate load indication, lack of primary stability, and late implant loss is associated with occlusal trauma, overloading and peri-implantitis [40,42,43].

Peri-implant pathologies represent the main cause of late dental implant failure [39]. Peri-implant infections are classified as peri-implant mucositis when induced inflammation is limited to soft tissues around the implant, or peri-implantitis if inflammation extends to the underlying bone [35]. Peri-implantitis is a pathological condition induced by plaque accumulation, affecting tissues around dental implants, and is characterized by peri-implant mucosa inflammation and progressive resorption of surrounding bone [39].

Other complications of dental implant surgery include maxillary sinusitis and displacement or migration of the implant into the maxillary sinus [44]. Odontogenic sinusitis typically occurs when the mucoperiosteum or Schneider membrane of the maxillary sinus is injured [45]. Odontogenic sinusitis induced by dental implants is often associated with dental procedures such as maxillary dental implant placement or sinus lift augmentation procedures, Schneiderian membrane of the maxillary sinus floor perforation, and displacement of implant or augmentation materials into the sinus cavity [44,45,46].

According to the included studies in this scoping review sinus graft infections are a major complication that can occur in the augmented bone area, requiring urgent treatment because of the risk of dissemination of the infection. As we can state after analyzing the cases presented in the included studies, sinusitis could appear after sinus graft contamination. Sinus graft infection could appear immediately after sinus lift intervention, due to intraoperative contamination, in this case, acute sinusitis occurred in few days after the intervention, or after a period in which peri-implantitis appeared around the inserted implants and contaminated the bone graft and this leads to sinusitis [4,6,7,10,31,32,34].

Untreated peri-implantitis could lead to major complications that could affect the patient’s quality of life. Our research uncovered a case of a patient in which peri-implantitis not only caused sinusitis but was also complicated by a brain abscess that caused the patient’s death [28].

These findings underline the importance and necessity of periodic reassessment of patients who have undergone bone augmentation surgery and insertion of dental implants, as well as the crucial role of oral hygiene in preventing these infectious complications that can threaten patients’ lives.

Once surgical dental treatments are developed, including dental implant insertion and bone augmentation, rates of iatrogenic cases of sinusitis are increasing. During these invasive treatments, the Schneiderian membrane of the sinus floor could be damaged. In the articles included in this review, we identified some cases of oro-antral communications both in zygomatic implants and conventional implants as well. Most cases of oro-antral communication appeared after the removal of dental implants affected by peri-implantitis. There were also cases of sinusitis induced by peri-implantitis, in which oro-antral communication occurred after implant removal, and sinusitis persists after communication closure [5,6,7,30].

Regarding bone graft materials extruded into the sinus cavity and implant migration into sinuses, studies in this review revealed that they were more frequent in implant-related odontogenic sinusitis sites compared to healthy control ones [4,29,31,34].

Zygomatic implants are used in cases with insufficient amount of bone tissue for anchoring conventional dental implants after advanced bone resorption, neoplasms, traumatisms, or heavily pneumatized maxillary sinus [5].

Zygomatic implants could produce biological problems such as sinusitis, soft tissue infections, oroantral fistula, chronic pain, facial nerve injury, infraorbital nerve damage, peri-orbital/facial hematoma [30]. The clinical manifestations of sinusitis starting from a zygomatic implant resemble other types of sinusitis and are characterized by nasal obstruction, hyposmia, halitosis, facial pain, congestion, dental pain, headache, and fatigue [5].

Although more investigations are necessary to demonstrate a straightforward cause-effect relationship between the placement of zygomatic implants and sino-nasal complications, these complications could occur because of the alteration of local homeostasis during the surgical insertion of these implants and the anatomical conditions of the patient [5].

The most frequent complication in zygomatic implants is sinusitis. Local infections, mucositis, or peri-implantitis are directly related to the occurrence of sinusitis and, also oro-antral communications produced by Schneiderian membrane perforation, lack of osteointegration of zygomatic implants along with functional forces may increase the risk of sinusitis development [47].

Our article emphasizes the existence of a correlation between peri-implant pathology and maxillary sinusitis. This condition is a common source of headaches as cited in the literature [9,11] and dental procedures such as bone augmentation, sinus lift, and implant placement might lead to sinusitis and nasosinusal headache [9,11,48].

There are some conditions that limit our review articles such as including only articles written in English and French, the fact that we find out in publications mostly case reports and case series, not large prospective or retrospective studies, the heterogeneity of the reported cases in the articles included in our review [48].

## 5. Conclusions

This scoping review revealed that there are many local conditions of dental implants that can produce sino-nasal complications such as maxillary sinusitis. Peri-implant infections, mucositis or peri-implantitis, oro-antral communications, or foreign body displacement into the sinus cavity could result in sinusitis. The conclusions of this literature review underlie the importance of respecting the insertion protocol of dental implants and minimizing the risks of injuries during the surgical step. The patient’s selection criteria are rigorously applied to avoid inserting implants in cases that are contraindicated. The importance of regular check-ups with the dentist after placing an implant and good oral hygiene maintenance to prevent infectious pathologies that can occur around implants are also important steps in preventing sinus diseases. Our review offers a starting point for future investigations on the correlation between peri-implant infectious diseases and maxillary sinusitis and we strongly recommend larger prospective studies on this topic. Further investigations could lead to improved protocols in implantology to avoid odontogenic sinusitis occurrence, better treatment strategies, and reduced complications in implantology.

## Figures and Tables

**Table 1:** Risk of bias assessment of case reports and case series studies. Questions 1–5: 1. Did the patient(s) represent the whole case(s) of the medical center? 2. Was the diagnosis correctly made? 3. Were other important diagnoses excluded? 4. Were all important data cited in the report? 5. Was the outcome correctly ascertained?

First Author/Year	No. of Cases	Question 1	Question 2	Question 3	Question 4	Question 5	Methodological Quality	Risk of Bias
Steiner, C./2021 [28]	1	YES	YES	YES	YES	YES	HIGH	LOW
Hong, S.O./2017 [10]	1	NO	YES	YES	YES	YES	MODERATE	MODERATE
D’Agostino, A./2021 [5]	42	YES	YES	YES	YES	YES	HIGH	LOW
Park, W. -B./2020 [6]	1	NO	YES	YES	YES	YES	MODERATE	MODERATE
van de Loo, S./2013 [29]	1	YES	YES	YES	YES	YES	HIGH	LOW
Scarano, A./2017 [7]	5	YES	YES	YES	YES	YES	HIGH	LOW
Yalçın, M./2020 [30]	45	NO	YES	YES	YES	YES	MODERATE	MODERATE
Park, W.-B./2019 [31]	124	NO	YES	YES	YES	YES	MODERATE	MODERATE
Rodriguez, A./2019 [32]	5	NO	YES	YES	YES	YES	MODERATE	MODERATE
Hainăroșie, R./2019 [4]	21	NO	YES	YES	YES	YES	MODERATE	MODERATE
Al-Juboori, M.J./2019 [33]	1	NO	YES	YES	YES	YES	MODERATE	MODERATE

**Table 2:** NOS scores for the included study.

Study	Selection	Comparability	Outcome	NOS Score	Methodological Quality	Risk of Bias
Case–control studies						
Park, M.J./2023 [34]	***	*	***	7	HIGH	LOW

NOS Scale for quality assessment of studies. The higher number of stars (*), the better quality. The maximum (*) possible are 9: **** for Selection, ** for Comparability and *** for Outcome.

**Table 3:** Study Characteristics.

First Author/Year	Study Design	Country	No. of Patients	Age/Mean Age of Patients	Gender of Patients	Associated Pathologies/Risk Factors	Type of Intervention	No. of Implants/Localization	Studied Pathologies	Follow-Up Length (Median)
Steiner, C./2021 [28]	Case report	Austria	1	62 years	Male	Not mentioned	dental implant insertion	1/in the left upper jaw	brain abscess in the left frontal lobe left maxillary and frontal sinusitis	10 months
Hong, S.O./2017 [10]	Case report	Republic of Korea	1	47 years	Male	Not mentioned	Sinus elevation, bone graft, and implant insertion 10 days previously	1/right upper jaw	sinusitis of the right maxilla	3.5 months
D’Agostino, A./2021 [5]	Case series	Italy	42	63 ± 10 years (range 29–81) years	16 males and 26 females	8 patients were smokers	Zygomatic implant insertion with 5 years follow-up	Upper jaw	Peri-implantitis, maxillary sinusitis, oroantral communication	60 months
Park, W. -B./2020 [6]	Case report	Republic of Korea	1	-	Male	No pathologies	lateral sinus augmentation simultaneously with implant placement, left posterior maxilla, 15 years ago	2 implants/incisor and premolar area	Peri-implantitis, oroantral communication, left maxillary, ethmoid, and frontal sinusitis	12 months
van de Loo, S./2013 [29]	Case report	Netherlands	1	65 years	Male	Not mentioned	Maxillary implants inserted 7 years ago	Upper jaw	Peri-implantitis, periodontitis, chronic maxillary sinusitis with recurring signs of acute sinusitis	4 months
Scarano, A./2017 [7]	Case series	Italy	5	54 years	3 males, 2 females	medical history of the patient was noncontributory	maxillary sub antral augmentation procedure and implant insertion	Posterior upper jaw	Peri-implantitis, maxillary sinusitis	3–6 years
Yalçın, M./2020 [30]	Case series	Turkey	45	51.76 years (range: 23 to 72) years.	24 males, 21 females	Patients with risk factor pathologies were excluded	Zygomatic implants insertion	Upper jaw	zygomatic implants with infection, peri-implantitis, sinusitis, oroantral fistula	17.2 months
Park, W.-B./2019 [31]	Case series	Korea	8	48.3 ± 7 years	7 males, 1 female	5 patients were smokers	Implant placement with sinus floor augmentation	Upper jaw	Peri-implantitis, Maxillary sinusitis	3 years
Rodriguez, A./2019 [32]	Case series	California	5	58.7 years	2 males, 3 females	1 patient with a medical history of angina, hypercholesterolemia, aortic valve stenosis, and periodontal disease, and another 1 with hypertension, hypercholesterolemia, osteoarthritis, asthma, and periodontitis	Bone augmentation procedures and dental implant insertion	Upper jaw	sinus and maxillary bone pathologies, displacement of the graft materials, oroantral communication, peri-implantitis, maxillary cysts	-
Hainăroșie, R./2019 [4]	Case series	Romania	21	47 years	7 males, 14 females	5 patients with diabetes mellitus	sinus lift procedure, concomitant with dental implant insertion	Upper jaw	Peri-implantitis, acute bacterial maxillary rhinosinusitis, acute fungal maxillary rhinosinusitis, implant migration into the maxillary sinus, orbital abscess, malar abscess	6 months
Al-Juboori, M. J./2019 [33]	Case report	Iraq	1	45 years	Female	No systemic diseases, no smoking	Implant insertion	Upper right lateral incisor	Chronic sinusitis with sinus discharge at the site of submerged implant	1 month
Park, M.J./2023 [34]	Case-control	South Korea	60	59.5 ± 19.1	32 males, 28 females	15 tobacco smokers and 28 social alcohol drinking	Implant insertion, bone graft	Upper maxilla	Peri-implantitis, maxillary sinusitis.	-

## Data Availability

Not applicable.
